# Predicting balance impairments in older adults: a wavelet-based center of pressure classification approach

**DOI:** 10.1186/s12938-023-01146-3

**Published:** 2023-08-22

**Authors:** Hedyeh Jafari, Thomas Gustafsson, Lars Nyberg, Ulrik Röijezon

**Affiliations:** 1 Department of Computer Science, Electrical and Space Engineering, Luleå University of Technology, Luleå, Sweden; 2 Department of Health, Education and Technology, Luleå University of Technology, Luleå, Sweden

**Keywords:** Balance, Wavelet analysis, Clustering, Classification, Sensorimotor, Ageing

## Abstract

**Background:**

Aging is associated with a decline in postural control and an increased risk of falls. The Center of Pressure (CoP) trajectory analysis is a commonly used method to assess balance. In this study, we proposed a new method to identify balance impairments in older adults by analyzing their CoP trajectory frequency components, sensory inputs, reaction time, motor functions, and Fall-related Concerns (FrC).

**Methods:**

The study includes 45 older adults aged $75.2 (\pm 4.5)$ years who were assessed for sensory and motor functions. FrC and postural control in a quiet stance with open and closed eyes on stable and unstable surfaces. A Discrete Wavelet Transform (DWT) was used to detect features in frequency scales, followed by the K-means algorithm to detect different clusters. The multinomial logistic model was used to identify and predict the association of each group with the sensorimotor tests and FrC.

**Results:**

The study results showed that by DWT, three distinct groups of subjects could be revealed. Group 2 exhibited the broadest use of frequency scales, less decline in sensorimotor functions, and lowest FrC. The study also found that a decline in sensorimotor functions and fall-related concern may cause individuals to rely on either very low-frequency scales (group 1) or higher-frequency scales (group 3) and that those who use lower-frequency scales (group 1) can manage their balance more successfully than group 3.

**Conclusions:**

Our study provides a new, cost-effective method for detecting balance impairments in older adults. This method can be used to identify people at risk and develop interventions and rehabilitation strategies to prevent falls in this population.

## Background

The postural control system plays a crucial role in maintaining the inherently unstable human body’s balance. This complex process integrates sensory input, primarily from vision, vestibular, and somatosensory systems, processes it within the Central Nervous System (CNS) and coordinates muscle activation to preserve stability and prevent falls [1]. The aging process is associated with the decline of these mechanisms and increases the risk of falls [2, 3]. In fact, falls present a substantial public health issue worldwide, impacting individuals across all age groups but particularly impacting older adults [4]. Therefore, understanding the aging effects on the postural sway mechanism and identifying and predicting balance impairments is an important issue that needs considerable attention.

To address this issue, it is crucial to understand the relationship between the sway generated by the CNS and the sensory information provided to the CNS in a closed-loop feedback system [5]. To study this relationship, posturography has been employed in numerous studies [6–10], which measure the trajectory of the individuals CoP by the force plate (statokinesigram). During a quiet stance, the CoP trajectories indicate the postural sway that occurs throughout the task, providing insights into an individual’s balance and postural stability [11]. In many research work, the CoP features, such as CoP ellipse area [12], path length [13], amplitude [14], the average CoP speed [15], the standard deviation, and Root Mean Square Error (RMSE) [16], are utilized in the time domain. Although the simplicity and the ease of interpretation in the time domain, it lacks to identify all oscillatory components of the sway and is less sensitive to subtle changes in postural sway [17].

As a result, some researchers have explored the frequency domain of the CoP in postural sway studies [18]. Various methods, such as fractional Brownian-motion analysis [19], slow (rambling) and fast (trembling) components [20], have been proposed to decompose the CoP signal into different components. These methods can reveal different aspects of postural control, and it has been argued that the slow component is in the sensory feedback loop while the fast component represents mechanical stiffness and motor commands [20, 21]. However, the literature has some variations and uncertainties regarding their interpretations and underlying mechanisms [22]. The Fourier transform is another technique used to analyze the CoP signal in the frequency domain [23, 24]. Although this method offers valuable insights into estimating power distribution within the frequency spectrum, it needs to provide information about various timescale corrections that can occur at different time instances. Since the CoP signal exhibits nonstationary characteristics and its frequency content changes over time, using this method should be approached with caution [25]. On the other hand, wavelet analysis is a method that transforms the time series signal into various time scales and frequency bands, making it suitable for intermittent, time-localized dynamics occurring in nonlinear systems with time delays [17].

### Related work

Analyzing the CoP signal and the relationship between frequency components and the decline in sensory systems was first studied in [17]. The authors utilized discrete wavelet analysis and discovered that older individuals exhibited reduced energy in longer timescales and increased energy in shorter timescales when vision was lost. This supports the idea that vision is used to control low frequency. However, the study had a small sample size. Moreover, it did not investigate the relationship with other sensory inputs, such as vestibular and proprioception, or fall risk factors like FrC. In ref. [26], authors used DWT for feature extraction of the CoP signal and discovered that the most critical information about postural sway was contained primarily in the lower frequency levels. However, there are variations concerning the cutoff frequency values and the exact mechanisms underlying the different frequencies [27]. Consecutive research has suggested a more detailed analysis of the frequency bands. While it must be amplified that there are significant variations and overlaps between studies, it has been suggested that approximately visual feedback is represented in $(< 0.1)~Hz$, vestibular in $(0.1-0.5)~ Hz$, cerebellum $(0.5-1)~Hz$ and somatosensory refluxes, motor commands and stiffing strategies in ($>1~Hz$) [25, 27–29]. This inconsistency highlights the need for further research and standardization in the field to understand better and interpret the frequency components of postural sway and their implications for balance and stability.

Recently, there has been a growing interest in using machine learning algorithms to predict balance impairments and falls [30]. By utilizing wavelet analysis and machine learning, the authors in [31] showed that somatosensory input changes have a vital role in postural control. In ref. [32, 33], they have used CoP signal and a classification algorithm to predict the risk of falls based on the history of falls. While fall history is essential to consider in balance impairment, other physical and psychological factors, such as FrC, also play a significant role in the postural sway of older adults [34]. Considering these factors when evaluating and addressing balance issues in this population is essential.

The impact of sensorimotor functions on CoP has been a primary focus of our lab [24]. Our research examined the CoP signal using the Power Spectral Density (PSD) of frequency domains in both eyes-open and eyes-closed trials. Our findings revealed a strong correlation between sensorimotor decline and higher FrC among individuals who could not adapt their balance strategies when vision was unavailable.

### Contributions

The primary objective of this article is to significantly advance our comprehension of the postural sway measures in older adults by investigating the CoP signal in a quiet stance. Furthermore, we aim to develop a prediction model that leverages sensorimotor decline and FrC to facilitate the early detection of balance impairment in older individuals. As a result, this work makes three notable contributions.

First, we carefully examine the CoP signal of older adults in challenging trials characterized by the absence of visual feedback and the presence of unstable surfaces. We employ wavelet analysis to achieve this, allowing us to explore the detailed changes of the CoP signal during these various conditions. By conducting such an in-depth investigation, we offer novel insights into the postural control mechanisms employed by older individuals, particularly when faced with situations that place higher demands on their balance abilities.

Second, we employ feature extraction techniques, specifically the discrete wavelet transform (DWT) and the k-means algorithm, to comprehensively cluster the CoP time series signal. This clustering approach allows us to identify distinct patterns and behaviors within the CoP data, helping us understand the underlying factors contributing to balance impairment in older adults. By outlining these patterns, we provide a framework for categorizing individuals based on their postural sway characteristics, which can have significant implications for personalized interventions and targeted treatment strategies.

Finally, we employ multinomial logistic regression to establish a predictive model elucidating the relationship between sensorimotor decline and FrC within the identified clusters. This modeling approach enables the identification of key predictors that can aid in the early identification of balance impairment in older individuals.

Overall, these contributions provide a comprehensive framework for investigating postural sway in older adults, offering novel insights into the underlying mechanisms and paving the way for the development of targeted interventions for the early detection and management of balance impairment.

## Results

**Fig. 1:**
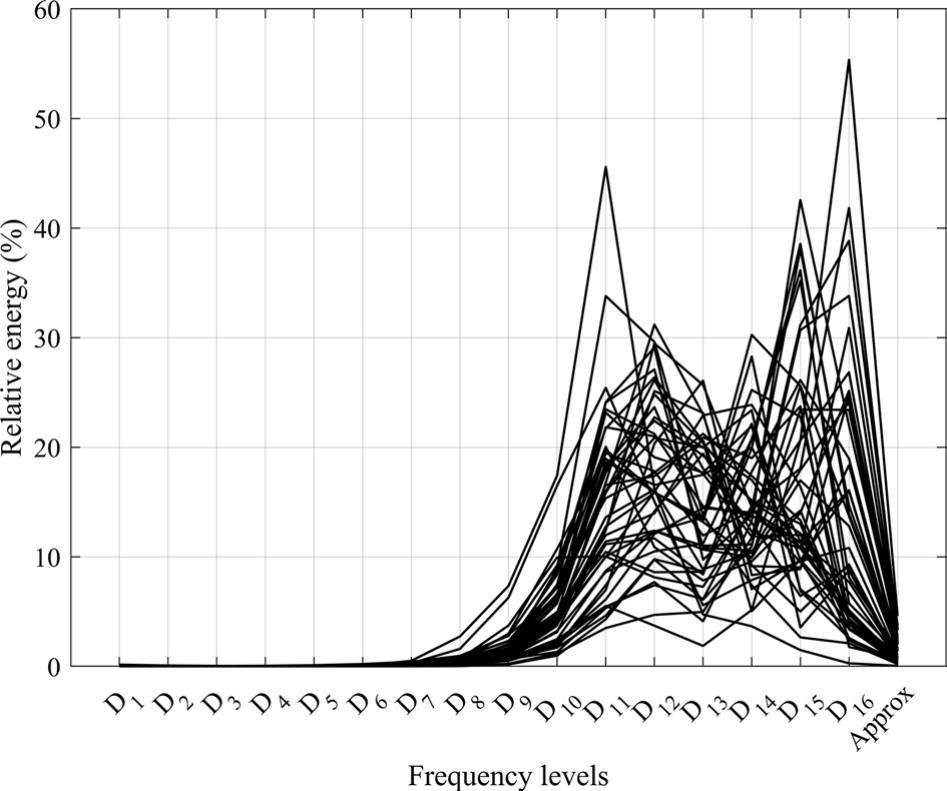
Relative energy of each decomposed frequency level of the center of pressure in stable surface with open eyes trial for all the subjects in the data set

As the CoP signal in quiet stance contains significant components at low frequencies, we discovered that a 16-level decomposition allows for the differentiation of low-frequency scale components based on their relative energy disturbance. Decomposing the signal to fewer than 16 levels only indicates that most of the signal’s energy is in the low-frequency range without providing specific information on how the energy is distributed across different low-frequency levels. Table 1 summarizes the frequency levels as well as the relative energy of each component. Figure 1 shows the relative energy of each frequency component for all subjects in Stable Eyes Open (SEO) trials. It can be seen that the majority of energy is concentrated in frequency levels

**Table 1:** Levels and relative energy of each component by discrete wavelet transform for all subjects’ center of pressure, in quite standing trial on a stable platform with eyes closed

Levels	Frequency (*Hz*)	Relative energy $(mean \pm sd )\%$
$D_1$	[750–1500]	0.025 ± 0.03
$D_2$	[338–832]	0.013 ± 0.01
$D_3$	[168–409]	0.009 ± 0.01
$D_4$	[84.1–204]	0.008 ± 0.01
$D_5$	[42.1–102]	0.013 ± 0.01
$D_6$	[21–51]	0.027 ± 0.03
$D_7$	[10.5–25.5]	0.089 ± 0.10
$D_8$	[5.26–12.7]	0.397 ± 0.45
$D_9$	[2.63–6.37]	1.571 ± 1.37
$D_{10}$	[1.31–3.19]	5.469 ± 3.49
$D_{11}$	[0.657–1.59]	15.184 ± 8.23
$D_{12}$	[0.329–0.797]	17.119 ± 7.10
$D_{13}$	[0.165–0.398]	14.047 ± 6.4
$D_{14}$	[0.086–0.199]	14.544 ± 6.22
$D_{15}$	[0.0488–0.099]	16.763 ± 10.46
$D_{16}$	[0.033–0.041]	13.347 ± 12.40
*Approx*	[0–0.00813]	1.367 ± 1.04

$(D_{10})$ to $(D_{16})$ [frequencies ($0.033 - 3.19~Hz$)], while other levels hold less significance.

**Fig. 2:**
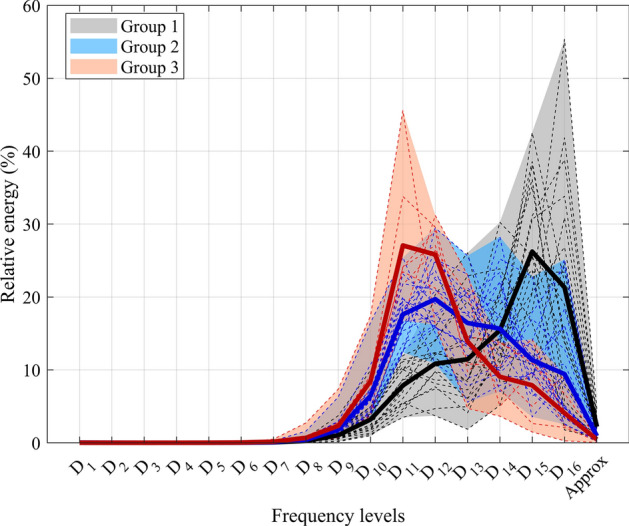
Three clustered groups illustrating relative energy distribution across frequency scales for the center of pressure signal during static standing on a stable surface with open eyes. Solid lines represent mean values; dashed lines indicate individual subjects’ energy values within each group, and shaded regions depict the energy range for each cluster

In order to cluster the data, we employed three groups to determine if the method could identify three categories of frequency levels: low, medium, and high. Figure 2 shows the result of clustering the relative energy distribution of each frequency component of SEO trial into three groups. It can be seen from the figure that three distinct groups can be detected successfully based on the distribution of relative energy across frequency scales. Group one (depicted in black–gray color) exhibits higher energy

($mean > 20 \%$) in very low-frequency scales [$D_{15}-D_{16} = (0.033-0.1) Hz$ ]. Group two (illustrated with blue color) demonstrates a normal distribution of energy across frequency scales, with relative energy between ($10\%< mean < 20 \%$) in the frequency range of [$D_{11}-D_{16} = (0.033-1.6) Hz$]. Group three (depicted in red color), in contrast, displays dominant energy ($mean > 20 \%$) in higher frequency scales [$D_{11}-D_{12} = (0.32-1.6) Hz$].

Table 2 summarizes the statistical analysis of the contribution of FrC, sensory inputs, reaction time, and muscle strength across different groups. The table demonstrates that the second group exhibits lower FrC, quicker reaction times, increased pressure sensitivity (particularly in the right foot), superior proprioception in all assessed joints, and greater muscle strength compared to the overall average of all participants. In contrast, group 3, displays higher FrC, slower reaction times, and diminished pressure sensitivity in the right foot. Moreover, a higher number of individuals in this group experienced falls in the past six months. Conversely, Group 1 is characterized by a significantly reduced sense of proprioception in the neck relative to the other groups.

**Table 2:** Descriptive value of each group’s fall-related concerns, sensory inputs, and muscle strength

Variables	Name	Group 1 ($n_1 = 18$)	Group 2 ($n_2 = 20$)	Group 3 ($n_3 = 7$)	All($n = 45$)
$x_1$	FES-I ($mean \pm sd$)	21 ± 4	19 ± 3	24 ± 7	21 ± 4.5
$x_2$	Reaction time(ms) $mean \pm sd$	387 ± 59	361 ± 76	416 ± 141	397 ± 106
$x_3$	Eyesight ($mean \pm sd$)	0.78 ± 0.13	0.72 ± 0.22	0.74 ± 0.17	0.75 ± 0.18
$x_4$	Touch sensation left foot (g)	3 ± 3.4	3.4 ± 3	3.25 ± 3.31	3.24 ± 3.14
$x_5$	Touch sensation right foot (g)	3.73 ± 3.57	3.4 ± 3.05	4.6 ± 3.8	3.75 ± 3.34
$x_6$	Neck proprioception left (degree)	4.6 ± 3.6	3.15 ± 3.6	3.75 ± 2.4	4 ± 3.15
$x_7$	Neck proprioception right (degree)	4.54 ± 3.79	3.28 ± 2.57	3.7 ± 2.27	3.97 ± 2.99
$x_8$	Knee proprioception left (degree)	7.25 ± 6.28	5.42 ± 5.91	6.48 ± 3.0	6.32 ± 5.68
$x_{9}$	Knee proprioception right (degree)	6.47 ± 5.12	5.08 ± 3.84	6.9 ± 3.43	5.6 ± 3.76
$x_{10}$	Ankle proprioception left (degree)	4.73 ± 3.9	4.05 ± 2.17	4.67 ± 2.12	4.61 ± 2.93
$x_{11}$	Ankle proprioception right (degree)	5.79 ± 5.05	4.39 ± 2.44	5.81 ± 3.9	5.13 ± 3.9
$x_{12}$	Hip extension left (N.m)	45.33 ± 18.46	49.43 ± 16.96	48.88 ± 20.11	47.29 ± 19.89
$x_{13}$	Hip extension right (N.m)	50.62 ± 22.84	53.14 ± 20.84	51.10 ± 21.52	51.68 ± 22.86
$x_{14}$	Hip abduction left (N.m)	48.22 ± 21.07	54.36 ± 22.4	51.36 ± 25.63	51.19 ± 23.73
$x_{15}$	Hip abduction right(N.m)	54.38 ± 22.93	58.06 ± 19.93	52.79 ± 31.16	55.53 ± 24.87
$x_{16}$	Knee extension left (N.m)	86.18 ± 26.15	89.5 ± 19.01	87.05 ± 29.71	86.61 ± 29.04
$x_{17}$	Knee extension right (N.m)	79.94 ± 26.51	87.88 ± 28.94	87.38 ± 25.87	84.25 ± 29.83
$x_{18}$	Knee flexion left (N.m)	66.08 ± 19.31	73.37 ± 27.54	67.86 ± 22.67	67.79 ± 24.51
$x_{19}$	Knee flexion right (N.m)	69.45 ± 22.51	76.46 ± 29.85	69.58 ± 24.28	70.45 ± 26.87
$x_{20}$	Ankle dorsal flexion left (N.m)	21.66 ± 5.39	26.15 ± 6.23	20.74 ± 9.1	21.79 ± 7.91
$x_{21}$	Ankle dorsal flexion right (N.m)	23.59 ± 6.8	24.24 ± 13.73	22.43 ± 7.8	23.01 ± 3.01
$x_{22}$	Ankle plantar flexion left (N.m)	83.89 ± 34.46	88.41 ± 32.71	83.34 ± 29.48	85.48 ± 35.23
$x_{23}$	Ankle plantar flexion right (N.m)	82.20 ± 26.27	86.75 ± 34.9	77.86 ± 26.60	81.79 ± 35.53
$x_{24}$	Falls history	33.3 $\%$	20$\%$	43$\%$	29$\%$

Tables 3 and 4 offer a more detailed understanding of the relationship between sensorimotor function, as shown by the multinomial logistic regression results. Table 3 presents the coefficients, parameters of the model, and the error of prediction according to (4) where the probability of being in group 1 versus group 3 is calculated, where Table 4 presents the model’s coefficient, parameters of the probability of being in group 2 versus 3, and the relative error of prediction. The small *p* value ($< 0.05$) of Falls Efficacy Scale-International (FES-I) FES-I ($x_1$), reaction time ($x_2$), touch sensation of both left and right foot ($x_4,x_5$), neck proprioception ($x_6$), knee proprioception ($x_8,x_{9}$), ankle proprioception of left foot ($x_{10}$) and hip muscle strength ($x_{13}$) indicates their significant contribution to the clustering of all groups. Eyesight ($x_3$) and knee muscle strength ($x_{16}$) also play crucial roles in distinguishing between group 2 and group 3. Furthermore, hip and ankle muscle strength $x_{14}, x_{15}, x_{22}$ are significant factors in determining the probability of an individual belonging to group 1 as opposed to group 3.

The results of this study reveal that individuals with better sensory input functionality, more efficient motor systems, faster reaction time, and fewer concerns about falls (group 2) tend to utilize a wide range of frequency scales of CoP during quiet standing (group 2). In contrast, subjects experiencing sensorimotor function decline and increased fall concerns either rely on very low-frequency scales (group 1) or higher-frequency scales (group 3) in their CoP usage.

**Table 3:** Model parameters for the probability of the center of pressure data being in group 1 versus group 3 based on the sensorimotor functions and fall-related concerns variables

Variables	*p* value	$\beta$ coefficients	Standard error of coefficient estimates	95$\%$ lower bound	95$\%$ upper bound
		36.86	34.46	− 30.7	104.4
$x_1$	0.0006$^*$	− 2.27	0.66	− 3.6	− 1
$x_2$	0.003$^*$	95.087	32.05	32.3	157.9
$x_3$	0.14	− 26.52	18.3	− 62.4	9.3
$x_4$	0.03$^*$	2.97	1.4	0.2	5.7
$x_5$	0.005$^*$	− 3.36	1.2	− 5.7	− 1
$x_6$	0.000003$^*$	4.9	1.06	2.8	7
$x_7$	0.04$^*$	1.26	0.67	− 0.1	2.6
$x_8$	0.002$^*$	1.73	0.56	0.6	2.8
$x_{9}$	0.0001$^*$	− 3.3	0.8	− 5	− 1.8
$x_{10}$	0.0001$^*$	− 3.98	1.07	− 6.1	− 1.9
$x_{11}$	0.27	− 0.84	0.77	− 2.4	0.7
$x_{12}$	0.61	− 23.21	46.25	− 113.9	67.4
$x_{13}$	0.03$^*$	− 151.64	71.1	− 291	− 12.3
$x_{14}$	0.0001$^*$	229.53	60.6	110.8	348.3
$x_{15}$	0.0004$^*$	− 199.43	56.4	− 310.1	− 88.8
$x_{16}$	0.12	67.11	44.1	− 19.4	153.6
$x_{17}$	0.06	− 87.52	46.6	− 178.9	3.9
$x_{18}$	0.727	− 13.88	39.78	− 91.9	64.1
$x_{19}$	0.998	0.06	42.4	− 83	83.2
$x_{20}$	0.4847	44.53	63.72	− 80.4	169.4
$x_{21}$	0.1565	82.89	58.5	− 31.8	197.5
$x_{22}$	0.04$^*$	− 118.48	57.7	− 231.6	− 5.4
$x_{23}$	0.39	39.84	46.5	− 51.3	131
$x_{24}$	0.12	8.33	5.36	− 2.1	18.9

$^*$ Significantly different (*p* value < 0.05)

**Table 4:** Model parameters for the probability of the center of pressure data being in group 2 versus group 3 based on the sensorimotor functions and fall-related concerns variables

Variables	*p* value	$\beta$ coefficients	Standard error of coefficient estimates	95$\%$ lower bound	95$\%$ upper bound
		114.3	41.3	33.5	195.1
$x_1$	0.00005$^*$	− 3.6	0.8	− 5.2	− 2.1
$x_2$	0.004$^*$	89.2	31.1	28.2	150.1
$x_3$	0.007$^*$	− 46.64	17.4	− 80.7	− 12.6
$x_4$	0.008 $^*$	3.8	1.43	1	6.6
$x_5$	0.005$^*$	− 3.8	1.34	− 6.4	− 1.2
$x_6$	0.001$^*$	3.3	0.9	1.3	5.2
$x_7$	0.83	− 0.16	0.8	− 1.7	1.4
$x_8$	0.004$^*$	1.3	0.45	0.4	2.2
$x_{9}$	0.00002$^*$	− 2.9	0.7	− 4.3	− 1.6
$x_{10}$	0.015$^*$	− 1.8	0.7	− 3.4	− 0.4
$x_{11}$	0.08	− 1.3	0.8	− 2.9	0.2
$x_{12}$	0.08	86.3	41.03	5.9	166.8
$x_{13}$	0.03$^*$	− 85.6	61	− 204.5	33.2
$x_{14}$	0.15	80.43	44.5	− 6.7	167.6
$x_{15}$	0.07	− 127.88	52.2	− 230.2	− 25.5
$x_{16}$	0.01$^*$	44.46	43.7	− 41.3	130.3
$x_{17}$	0.30	− 25.97	50.11	− 124.2	72.2
$x_{18}$	0.60	− 62.54	42.7	− 146.2	21.1
$x_{19}$	0.14	− 7.10	47.2	− 99.7	85.5
$x_{20}$	0.88	67.14	63	− 56.3	190.5
$x_{21}$	0.11	91.6	58.15	− 22.4	205.6
$x_{22}$	0.2	− 71.3	54.6	− 178.3	35.7
$x_{23}$	0.37	− 41.5	46.16	− 132	48.9
$x_{24}$	0.6	3.06	6.1	− 8.9	15

$^*$ Significantly different (*p* value < 0.05)

## Discussion

The study’s findings suggest that while both group 1 and group 3 exhibit declines in sensorimotor functions and increased fall concerns, group 1 demonstrates less sensorimotor function decline and fewer fall concerns than group 3. Notably, subjects unable to complete all trials belong to group 3, as discussed further below. This finding can help address the ambiguities in the literature regarding whether balance impairment occurs in higher [17, 35] or lower frequency scales [24]. Our results indicate that both scales can be linked to balance impairment, although individuals who utilize lower frequency strategies seem to maintain balance more successfully than those who rely on higher frequency scales. The most effective balance strategy (group 2) also utilizes a normally distributed range of high to low-frequency scales.

**Fig. 3:**
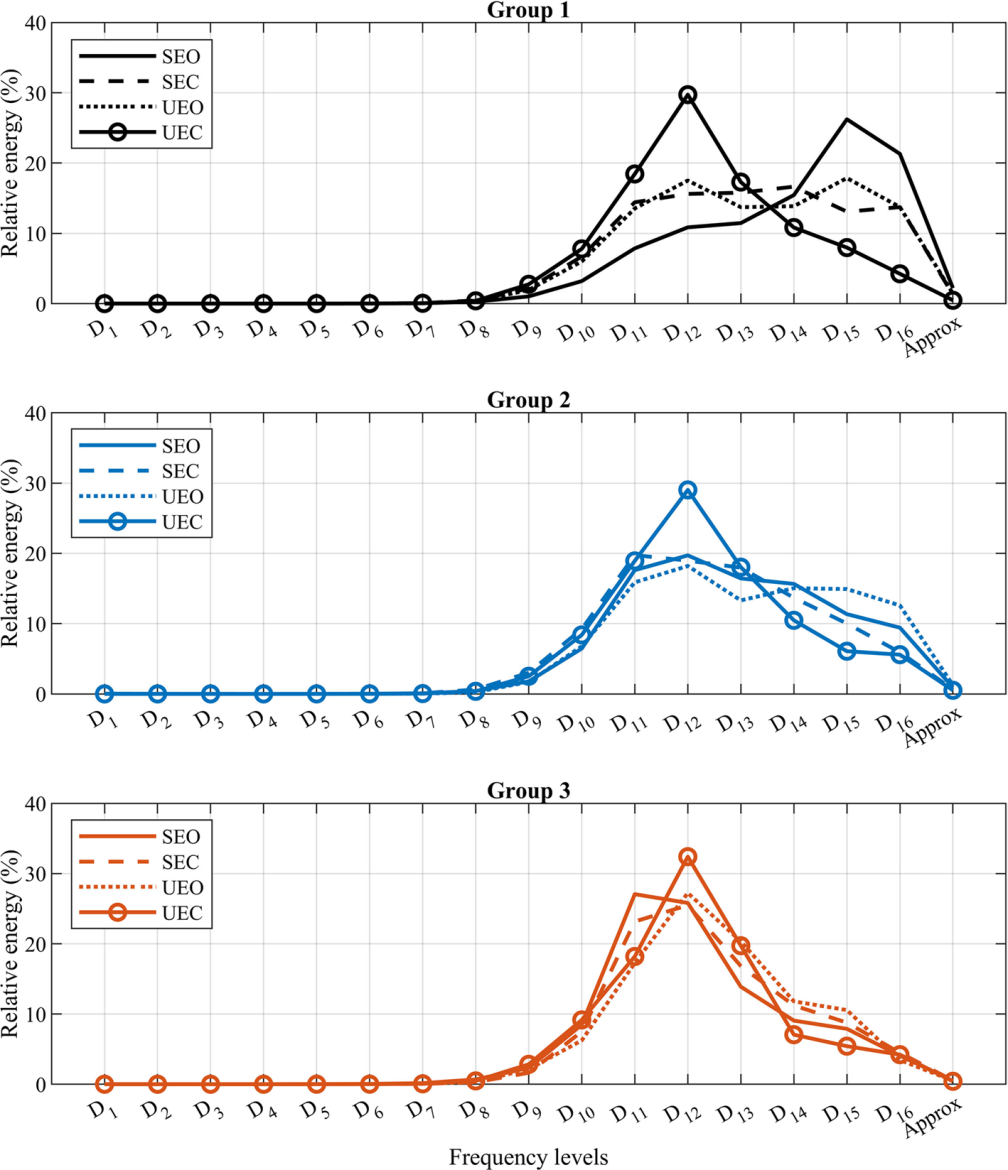
Mean value of relative energy of different frequency scales of CoP trajectory for three different groups in the trials of SEO (solid line), SEC (dashed line), UEO (dotted line) and UEC (solid-circle line)

Figure 3 shows the response of each group in relative energy of wavelet decomposition to the more challenging trials of Stable Eyes closed (SEC),Unstable Eyes Open (UEO), and Unstable Eyes Closed (UEC). Groups 1 and 2 changed to a more high-frequency strategy in case of challenging trials, while smaller adaptations were seen for group 3. Interestingly, all subjects in the data set use the same balance strategy of frequency usage in the most challenging trials of UEC with the dominant frequency level

$D_{12}$ [0.329$-$0.797] *Hz*. This suggests a common approach to maintaining balance in the face of extreme difficulty. Groups 1 and 2, on average, decreased the energy usage in lower frequencies level and increased the usage of the higher frequency levels from losing vision and standing on an unstable surface. This is in line with previous research showing increased usage of higher frequencies due to more challenging tasks [36]. As in many literature, lower frequencies of CoP are related to visual feedback [27]; this means this group of subjects rely more on vision, and in case of vision loss, they search for other feedback sensorimotor functions.

Group 2, on the other hand, decreases its usage of lower frequency levels when closing its eyes on a stable surface and decreases it even more on an unstable surface. In contrast, group 3 seems to exhibit a different strategy altogether, with a slight change in frequency usage towards lower frequency levels, and mainly usages higher frequency levels with the dominant frequency levels $D_{11}, D_{12}$ [0.329$-$1.6] *Hz* for all trials. Considering that these frequency bands are argued to mainly to vestibular and cerebellar functions [27], subjects in group 3 may rely more on these systems rather than visual feedback. Another interpretation could be that change toward higher frequencies is related to a stiffening strategy that increases muscle co-contractions [37], however, stiffing is argued to be in the even higher frequencies ($> 1~Hz$). While the current study observed balance impairments and their relationship to the decline in sensorimotor function and fall concerns in different groups, the investigation of the relationship between frequency scales and specific neural systems was not within the scope of this study. Therefore, any hypotheses regarding the neural mechanisms underlying the observed balance impairments in different groups should be considered preliminary, and further research would be necessary to confirm these hypotheses.

**Fig. 4:**
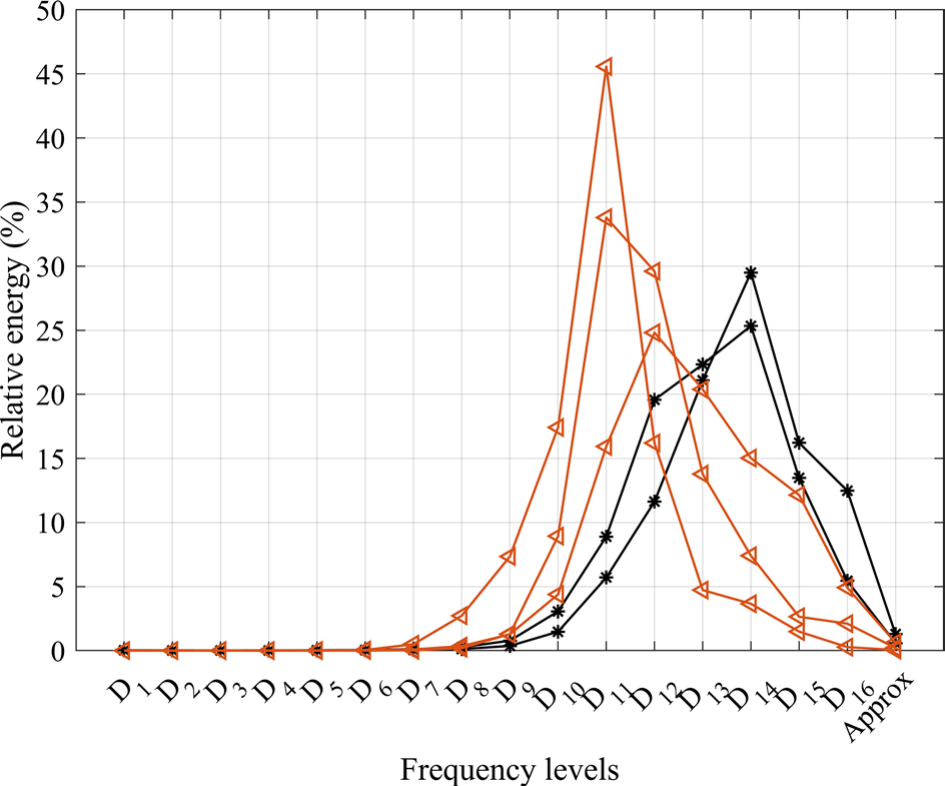
Relative energy of different frequency scales of CoP trajectory in SEO trial for healthy young subjects (black solid-star lines) and older adults who could not continue challenging trials (red solid-triangle line)

To validate our findings, we compared the CoP trajectory of two healthy young subjects (29 years old) in SEO trials with the subjects who were unable to complete the more challenging trials of UEO and UEC. Figure 4 presents the relative energy of each frequency level for these individuals. As depicted in the figure, the healthy young subjects belong to group 2, while those who were unable to continue the challenging trials belong to group 3. This indicates that group 2 seems to have the strategy of usage of frequencies similar to younger adults. On the other hand, aging appears to lead to a shift towards using higher frequencies (as observed in group 3) or lower frequencies (as observed in group 1) during balance control. Group 3 showed a higher incidence of falls in the past six months and difficulty completing postural control trials compared to group 1. This suggests that group 1 may have a more successful strategy for reweighing sensorimotor information and maintaining balance compared to group 3.

### Limitation and future direction

Although our proposed methodology provides valuable results in distinguishing the balance impairment in older adults, several limitations should be considered. First, a larger population of data is needed to guarantee the relationship between sensorimotor and CoP and generate our predictive model. Furthermore, it is essential to have a sensitive test for the vestibular input. Third, with a larger sample size, other prediction methods can be used to find a more accurate model. Finally, a more comprehensive follow-up study is needed to investigate the effect of intervention and rehabilitation studies on the sensorimotor functions that are significantly different in the groups to see if the frequency strategy will change among groups.

## Conclusions

This study aimed to enhance our understanding of the CoP signal in the postural sway of older adults and develop a prediction model based on sensorimotor functions decline and FrC that can be used for the early detection of balance impairment in older individuals. Our results revealed that wavelet decomposition’s relative energy could provide valuable insights into balance behavior. We identified three distinct cluster groups with differing balance behaviors. Our findings suggest that individuals with better sensorimotor functions and fewer concerns regarding falls utilized a wider range of frequency scales. Conversely, those with sensorimotor decline and fall-related concerns may use either very low-frequency scales or higher-frequency scales, and those using lower-frequency scales can manage their balance more successfully. Overall, our study presents a cost-effective approach to detecting balance impairments in older adults, and the predictive model can be used to develop interventions and rehabilitation strategies to prevent falls.

## Methods

Informed written consent was secured from every participant involved in the research. The study’s design received approval from the Umeå Regional Ethical Review Board in Sweden (reference number 2015-182-31), and it adhered to the principles outlined in the 1964 Helsinki Declaration.

### Sample

This study is part of the BAHRT (Balancing Human and Robot) project, in which participants were recruited from a community in Northern Sweden. Exclusion criteria for this study included having an MMSE (Mini-Mental State Examination) score of 23 or below, which indicates a level of cognitive decline that makes it difficult to follow instructions, being unable to complete the walking task in the Short Physical Performance Battery, and being unable to read large print (80pts block letters) in the MMSE. The analysis included 45 participants, comprising 27 women and 18 men, with an average age of $75.2~(\pm 4.5)$ years. Table 5 summarizes the characteristics of the participants.

**Table 5:** Characteristics of the participants

Characteristics (mean ± sd)	All (*n* =45)	Women (*n* = 27)	Men (*n* = 18)
Age	75.2 ± 4.5	76.0 ± 5.0	73.9 ± 3.3
Height (cm)	167.33 ±9.9	161.78 ± 9.6	176.47 ± 8.9
BMI	26.07 ± 3.76	26.05 ± 3.1	26.10 ± 2.8

### Data collection

Postural behavior was assessed during quiet stance by a force plate (Kistler, Switzerland) sampling at 3000 *Hz* across four distinct 30-s test scenarios: (1) stable (rigid) surface with open eyes: SEO, (2) stable surface with eyes closed: SEC, (3) unstable (soft) surface with eyes open: UEO, and (4) unstable surface with closed eyes: UEC. To standardize foot placement, each test was conducted with feet side by side and the first metatarsal heads at a distance equal to $75\%$ of the width between the anterior superior iliac spines, with a self-chosen rotational angle of the foot placement. Participants were instructed to stand up straight, focus on a dot on the wall, and remain as still as possible throughout the test. For the eyes-closed trials, participants first looked at the dot on the wall before closing their eyes. A trigger button was used to set a marker in the measurement to indicate the test’s initiation when the eyes were closed, and the posture was stable.

Sensorimotor such as eyesight, touch sensation, reaction time, proprioception of the neck, knee, and ankle joints, as well as strength of lower limb muscles of each participant was measured in our laboratory in a comprehensive protocol described by details in [38].

FrC was measured by FES-I instrument. The FES-I assesses an individual’s level of concern about falling while performing various tasks and has been proven to be a valid and reliable tool for this purpose. Scores range from 16 to 64, with higher scores indicating greater concern about falling [39].

### Structural design

**Fig. 5:**
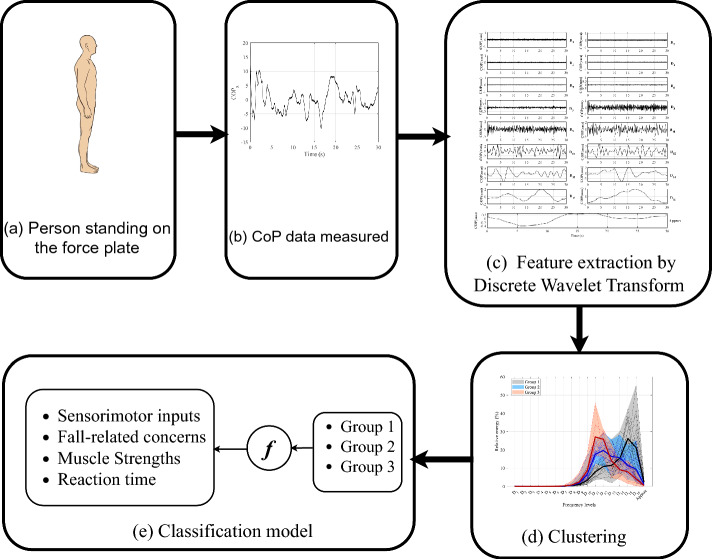
Diagram of the proposed structure to detect different groups of subjects based on the CoP trajectory and identified sensory contributions. **a** A subject will stand as still as possible on the force plate. **b** the CoP trajectory is measured in different trials of standing on stable and unstable surfaces with eyes open and closed **c** the data is filtered, and by DWT, features of the signal are extracted. By the k-means algorithm, the data of all participants are clustered into three groups. **d** based on the sensorimotor functions, FrC and their balance performance, and with utilizing the multinomial logistic classification method, the relationship between each group of subjects and their decline in sensorimotor functions and balance performance is detected

Figure 5 illustrates the diagram of the proposed method. First, raw data of CoP of the subjects are preprocessed. The data are detrended and filtered with Butterworth low pass filter with a 10 *HZ* cutoff. Second, in the feature extraction phase, the DWT separates each time-series signal into multiple frequency components with their own relative energy. Third, the k-means clustering algorithm is used to identify distinct groups within the data. After analyzing these groups, they are labeled accordingly. Finally, a multinomial logistic model is utilized to determine the contribution of each group to sensorimotor functions and FrC, as well as to predict the future signal. Algorithm 1 presents the overall algorithm of the presented method.

In this study, as the movements in the sagittal plane are predominant during a quiet stance, only the anterior–posterior direction of the CoP signal is utilized. All participants (45 individuals) were able to complete the SEO trial, whereas in the more balance challenging trials of (SEC, UEO and UEC) three subjects could not perform the trial successfully. We have discussed their balance behavior in the discussion section. It is important to note that we exclusively used SEO data for clustering and developing a model to predict balance. Our aim is to demonstrate that, in the future, an affordable and straightforward posturography test could be employed to predict balance impairments. Nonetheless, we utilized other challenging trials SEC, UEO, and UEC to analyze and validate our findings.


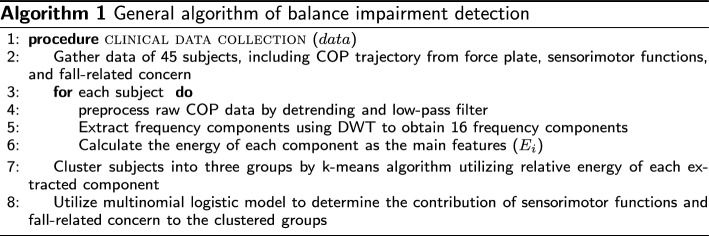


#### Features extraction by DWT

By maximal overlap DWT the preprocessed signal is decomposed into different signal components at different timescale resolutions or equivalently into different frequency bands. Each component has relative energy, representing that frequency band’s importance in the original signal [40].

**Fig. 6:**
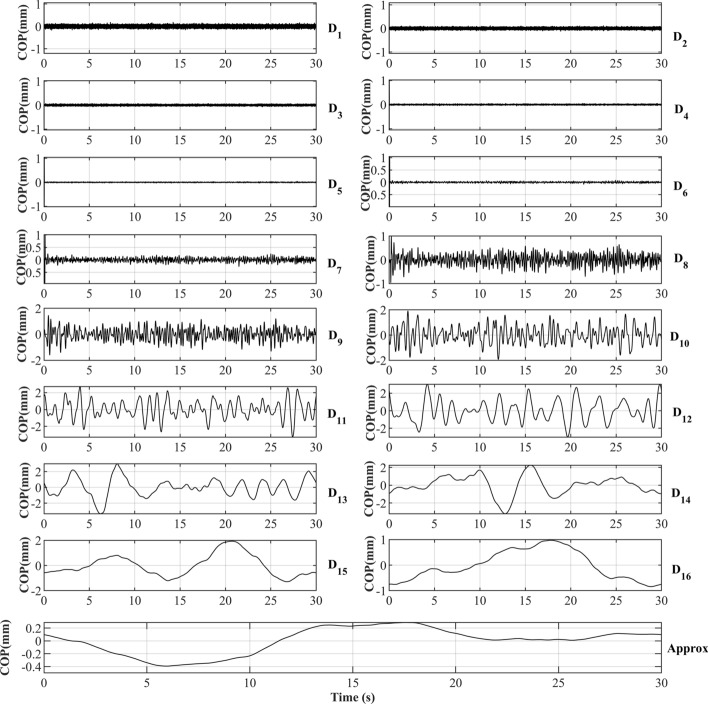
A 16-level discrete wavelet transform decomposition of a random subject’s center of pressure signal in the sagittal plane in standing on a stable surface with open eyes

The wavelet decomposition process includes two digital filters: low-pass or high-pass filters. The first level of the DWT can be described as follows:

1 $$\begin{aligned} A[n]= & {} (x * h)[n] = \sum _k x[k] \cdot h[n - 2k] \end{aligned}$$

2 $$\begin{aligned} D[n]= & {} (x * g)[n] = \sum _k x[k] \cdot g[n - 2k] \end{aligned}$$

where *x*[*n*] represent the origin signal, *h*[*n*] denotes the low pass filter coefficient and *g*[*n*] signifies the high pass filter coefficient. The first equation, which calculates the approximation coefficients, is associated with the low-frequency components of the signal. On the other hand, the second equation computes the detail coefficients, capturing the high-frequency components [41]. Later, the relative energy of each component at each frequency level ($i=1, \dots ,k$) can be calculated as

3 $$\begin{aligned} E_i\% = \frac{\sum _{j} (c_{ij})^2}{\sum _{k} \sum _{j} (c_{ij})^2}100\% \end{aligned}$$

where *c* notes all decomposed frequency components including details and approximation, *j* represents discrete CoP location. Figure 6 shows the DWT decomposition of a random subject’s CoP signal and Algorithm 2 describes the implementation process.


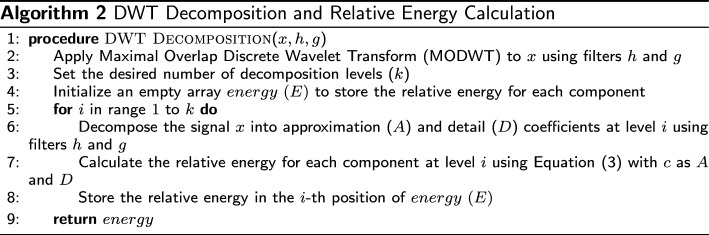

#### Clustering

The relative energy of each frequencies components is then used for clustering the subjects into different groups. Here, we used a k-means clustering algorithm for its simplicity and scalability of clustering matrices. K-means is a widely used unsupervised learning algorithm designed for partitioning a data set into distinct groups or clusters based on the similarity between data points [42]. The algorithm operates on a matrix of data, where in our case, the rows represent the observation of 45 subjects, and each column corresponds to the relative energy of each component as the features. K-means aims to minimize the within-cluster sum of squares (WCSS), which is the sum of squared distances between each data point and the centroid of the cluster it belongs. To achieve this, the algorithm initializes K centroids randomly or through a predetermined method, then iteratively refines these centroids by assigning each data point to the nearest centroid and updating the centroid as the mean of all points in the cluster. This process continues until the centroids converge, partitioning the data matrix into K homogeneous clusters [43]. The implementation algorithm is presented in the following.


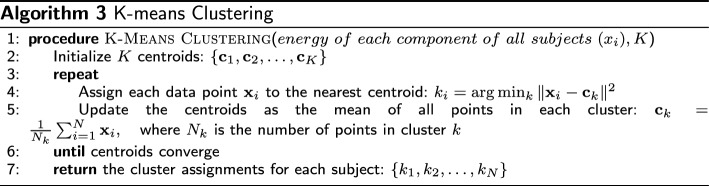


#### Multinomial logistic model

To analyze the obtained cluster, it is essential to understand the relationship between these groups and the sensorimotor and FrC. To achieve this, we employed the multinomial logistic model to find the interaction of sensorimotor and FrC with the different clusters, providing valuable insights into the underlying neural processes. Moreover, the resulting model was a robust prediction tool for future posturography signals.

Multinomial logistic regression is an extension of binary logistic regression used for predicting outcomes of categorical dependent variables with more than two classes [44]. It estimates the probabilities of each class by modeling the relationship between a set of predictor variables and a categorical outcome. The algorithm uses a series of binary logistic regression models, one for each class, with a common reference category.

The basic equation for multinomial logistic regression can be expressed as

4 $$\begin{aligned} P(Y_i = k) = \frac{e^{\beta _{k0} + \beta _{k1}X_{i1} + \cdots + \beta _{kp}X_{ip}}}{\sum _{j=1}^K e^{\beta _{j0} + \beta _{j1}X_{i1} + \cdots + \beta _{jp}X_{ip}}} \end{aligned}$$

where $P(Y_i = k)$ denotes the probability of the *i*-th observation belonging to class *k*, $X_{ij}$ represents the value of predictor *j* for observation *i*, $\beta _{kj}$ are the coefficients corresponding to predictor *j* for class *k*, and *K* is the total number of classes. Multinomial logistic regression offers robust implementation due to its lack of requirements for normality or linearity in the data. This flexibility enables the model to handle various types of relationships and data distributions effectively, making it a versatile choice for many classification tasks [45]. The implementation algorithm is presented in the following:
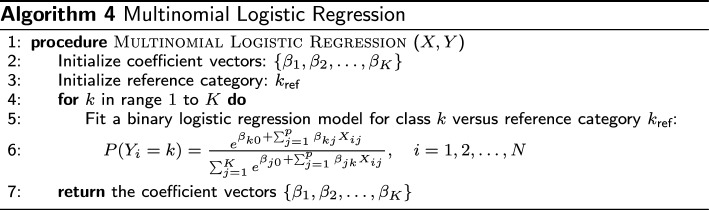


Feature selection is performed using MATLAB’s “modwt” function. Meanwhile, the “kmedoids” function in MATLAB is employed for clustering, and the “mnrfit” function is utilized for logistic regression analysis.

## Data Availability

Data cannot be shared publicly because it contains sensitive data including health status, anthropometrics, age and location (recruited from a relatively small community). Data are available from the LTU Institutional Data Access officer: Johan Lundberg Karlsson (contact via dataskydd@ltu.se) for researchers who meet the criteria for access to confidential data.

## Abbreviations

CNS: Central nervous system
CoP: Center of pressure
RMSE: Root mean square error
PSD: Power spectral density
FrC: Fall-related concerns
DWT: Discrete wavelet transform
SEO: Stable eyes open
SEC: Stable eyes closed
UEO: Unstable eyes open
UEC: Unstable eyes closed
FES-I: Falls efficacy scale-international
